# Supplementary material for: Are carbon emissions trading and green financial instruments synergistic? -Comprehensive quantitative research based on content analysis
Source: PLoS One. 2024 Mar 7;19(3):e0298601. doi: 10.1371/journal.pone.0298601 (PMC10919601; doi:10.1371/journal.pone.0298601)
Supplement: S1 Data — (ZIP) [file pone.0298601.s001.zip › Data/地级市Z_F工作报告.zip!/║■─╧/╓Ω╓▐╩╨/└┤╫╘╣1⁄2╓┌║┼í╛┬φ┐╦╩2╛▌═°í┐_2004.docx]

各位代表：

　　现在，我代表市人民政府向大会作工作报告，请予审查，并请市政协各位委员和其他列席同志提出意见。

　　一、紧扣第一要务，2003年工作目标全面实现

　　2003年，我们在市委的领导下，深入实践“三个代表”重要思想，全面贯彻党的十六大精神，紧扣发展第一要务，认真履行政府职责，圆满完成了市十二届人大一次会议确定的各项工作任务。全市实现国内生产总值390亿元，比上年增长11%；完成财政总收入27.9亿元，增长15.6%，其中一般预算收入16.8亿元，增长13.2%；城镇居民人均可支配收入达到8718元，增长10.6%，农民人均纯收入达到3010元，增长5%。

　　——突出三大工作重点，全面加快经济发展

　　按照市委九届八次全会精神，我们突出了深化国有企业改革、发展非公有制经济、扩大对外开放三个经济工作重点，促进了全市经济快速健康发展。

　　深入推进国有企业改革。市政府决定用两年左右的时间，以“置换国有资产，有偿解除职工劳动关系”为主要形式，基本完成市属国有企业改革任务。为顺利推进改革，市政府在财力十分有限的情况下，以社保挂帐、土地出让金返回等形式，承担改革成本近10亿元。当年确定的市属100户改制企业，前两批改制的38户工业企业全部召开了职代会，通过了改制方案，其中机床齿轮厂、株塑公司、锻压机床厂等12户企业的改制工作已全面完成，另有20户企业与战略投资者达成了收购意向，预计新增投入20亿元以上；确定的30家商贸改制企业已有19家完成了改革任务；建安等其他类型的32户企业也有2/3实现了改制重组。县(市)区属国有企业改革现已进入扫尾阶段,在株省、部属国有企业改革加快推进。

　　大力发展非公有制经济。市委、市政府研究出台了《加快非公有制经济发展的若干意见》，召开了全市发展非公经济大会，举办了中国·株洲炎帝节民营经济高峰论坛，市政府有关职能部门分别制订了实施细则。为支持非公经济发展，市财政注入小额贷款担保资金1500万元。通过努力，非公经济已成为全市经济增长的一支生力军，全年完成增加值157.9亿元，占GDP的比重达到40.5%。

　　不断扩大对外开放。市政府制定了《株洲市利用外资奖励办法》，建立了招商引资目标责任制。大力拓宽招商引资渠道，积极组织各类企业参加“温洽会”、“深洽会”等招商引资活动，先后召开广州、北京株洲籍人士座谈会，提高了株洲的对外开放度。全年实际利用外资8800万美元，引进内资28亿元，分别增长23.5%、40%。注重加大项目建设力度，以项目引资金，共向中央和省财政争取各类专项资金6.5亿元，其中国债资金2.4亿元。大力调整出口企业、出口产品和出口市场结构，进一步拓展了国际市场。全年完成进出口总额4.7亿美元，增长23.7%，其中出口3.5亿美元，增长14.8%。

　　重点工作的突破，推动了经济全面快速发展。农业结构调整稳步推进。全年完成农业总产值78亿元、农业增加值51亿元，均比上年增长3.5%；农业内部结构进一步优化，养殖业占农业的比重突破了50%；竹木、肉食、油脂、果蔬、优质米五大优势产业进一步壮大，农产品加工企业发展到1500多家，实现总产值近30亿元；乡镇企业保持良好发展势头，完成增加值144.5亿元，增长12%；农村税费改革成果进一步巩固，农民人平负担降到50元。工业经济运行质量和效益继续提高。规模以上工业完成增加值90亿元，增长18%，实现净盈利超过10亿元，增长40%，完成技改投入32亿元，增长20%。高新技术产业实现产值145亿元，增长13%。第三产业增长迅速。芦淞市场群25个市场的提质改造共投入资金6.2亿元，炎帝节服饰商务周取得圆满成功；旅游业发展步伐加快，全年实现旅游收入23.6亿元，增长16.2%；房地产业高速发展，完成交易额20多亿元，增长43%。现代物流、信息、交通、邮政、电信、金融、保险、证券、咨询业有了较快发展。城乡市场繁荣有序，实现社会消费品零售总额140亿元，增长10.5%。一、二、三次产业比例从2002年底的14.9：48.5：36.6调整为去年底的12.8：50.1：37.1。

　　——突出基础设施建设，致力构筑发展平台

　　狠抓城市基础设施建设，城市功能不断完善。加快城市建设投融资体制改革步伐，组建了城市建设投资经营有限公司，与国家开发银行签订了35亿元的金融合作协议，加快建设了一批重点工程。城市道路建设稳步推进。城市快速环道累计完成投资9亿元，开工总里程近20公里，到2005年可望全线贯通；创立了“市投区建、区投区建”的新模式，投入13亿元，相继完成了建设北路、红旗西路、田林路等7条城区主次干道和4条城区样板街的改造美化任务。园林城市创建步伐加快。动工兴建荷塘、天池两个开放式公园，建成了新华桥头绿地、红港路绿化休闲广场，城市人均公园绿地面积提高到7.9平方米，顺利通过了省级园林城市验收；南郊垃圾场一期工程完工并投入使用；建宁港整治项目和龙泉污水处理厂正在紧张地施工建设。规划龙头地位不断强化。第六次城市总体规划修编已经省政府批准，并报国务院审批；完成了市中心区4.5平方公里控制性详细规划；继续开展全市拆违大行动，拆除违法建设50万平方米。旧城改造取得新进展。通过招商引资，启动了“中国城”、中心广场西南角、中鸿人和广场等改造项目。

　　加强农村基础设施建设，农村生产生活条件得到改善。农村小城镇建设呈现新景象。共投入资金3.5亿元，加大了以县城提质扩容为重点的小城镇建设力度，全市城镇化水平达到40.8%。农田水利建设掀起新高潮。筹资3.2亿元，建设各类水利工程5万多处，重点治理了100座病险水库，完成了235公里大中型灌区渠道的防渗加固改造，以及城区8公里湘江防洪大堤和县城部分防洪堤建设，再次捧回全省水利竞赛“芙蓉杯”；株洲航电枢纽工程建设步伐加快，已累计完成投资4.8亿元。农村生态体系建设取得新成效。林业建设创历史最好水平，完成工程造林25.3万亩，其中退耕还林24万亩；多渠道投入资金近1亿元，继续推进了优质稻基地、农业综合开发项目的建设。县乡工业园区建设步伐加快。

　　积极推进交通能源建设，发展瓶颈逐步缓解。衡炎高速公路工可报告已通过评审，基本完成了初步设计；醴潭高速公路拆迁工作顺利结束；田黄高速公路预工可报告和工程评估已经完成；106国道全线提质改造继续推进，茶陵段22公里路段竣工交付使用，醴陵北段32公里已基本完成路基工程；莲易高等级公路株洲段改造进展顺利；县到乡（镇）公路建设稳步推进，村组道路建设来势喜人。“天然气入株”取得实质性进展；城市供水管网改造已经启动；株洲电厂4号机组正式投产；基本完成了农网二期改造，县城电网改造也已正式启动；加大了以小水电、沼气为重点的农村能源开发建设力度，改善了农村能源结构。

　　——突出以人为本理念，努力促进社会进步

　　积极应对“非典”挑战，卫生体系建设力度加大。我们按照“层层设防、群防群控、科学有序、严防死守”的工作方针，成功打赢了一场“非典”阻击战，全市无1例原发性病例。投入资金1120万元，建立并完善了多层次、高效率的疾病防治体系；争取市疾控中心与五县（市）防疫站分别被列入国家和省里的公共卫生建设项目，23所中心卫生院被列入省重点建设项目。

　　着力推进科技进步，科技创新能力有所增强。坚持把科技创新作为发展第一推动力的理念不变，把高新区作为全市重点发展区域的政策不变，高新区形成了“一区三园三基地”的发展格局。坚持利用高新技术和先进适用技术改造传统产业的思路不变，着眼于把株洲建成全省先进制造业基地，新增了1家博士后工作站，在全市61家大中型企业都建立了技术中心，科技创新能力进一步增强。时代集团承担的“863”计划科研项目取得突破，电动公交汽车试制成功。紧密结合招商活动，开展国际科技合作，全年共争取政府间科技合作项目和“双引”项目4项。

　　致力提升株洲形象，各项社会事业全面发展。教育方面，制定出台《株洲教育改革发展规划》，推进了教学和办学体制改革，炎陵县九年一贯制集中办学模式获得教育部、财政部肯定和资金支持；启动了湖南工业大学筹建工作；市一中等四所中学晋升为省重点中学。文化方面，承办了2003年湖南艺术节，创作了《工人组歌》、《红红的中国结》等一批好歌剧、好歌曲，《沥沥太阳雨》获全国“五个一工程奖”；新闻出版工作得到加强。体育方面，大力开展群众体育运动，举行了全市首届社区运动会；承办了第五届全国城运会武术比赛项目；按照产业化、市场化的运作模式，成功举办了中美女篮国际对抗赛、株洲高科杯首届全国汽车场地越野赛。广电、档案、市志、老龄、知识产权、民族宗教等事业和民兵预备役工作都有新的发展。

　　坚持可持续发展战略，人与自然发展更加和谐。认真贯彻《人口与计划生育法》，努力稳定低生育水平，人口自然增长率为3.7‰，继续保持全省领先水平；认真贯彻2001—2010年全市妇女儿童发展规划，妇女儿童事业持续发展；切实保护耕地，连续6年实现耕地占补平衡；严格国土与矿产资源开采管理，有效遏制了破坏生态的行为；切实加强植树造林、荒山治理和水土保持工作，森林覆盖率提高到56.6%；以清水塘地区为重点，加大了城市大气污染和水环境综合整治力度。

　　始终关心群众生活，社会保障工作成效明显。通过发展经济，拓展就业空间，新增就业岗位4.6万个；认真落实再就业优惠政策，加强就业技能培训，帮助3.6万名下岗失业人员实现再就业，城镇登记失业率为4.6%；大力组织劳务输出，全市在外务工人员达到45万人，劳务收入达到31亿元。认真做好社会保障工作，按时足额社会化发放养老金6.3亿元，下岗职工基本生活费1.1亿元，失业保险金2929万元，城镇“低保”户“低保”金5640万元，国企下岗职工和城镇“低保”户的基本生活得到了保障。基本完成了住房公积金管理机构的调整任务，累计归集住房公积金9.1亿元，当年发放职工个人住房贷款9600多万元。残疾人福利事业有了新的发展，扶贫开发工作取得了新成绩。

　　——突出政府职能转变，切实加强自身建设

　　致力建设有限政府。按照市场经济的要求，积极调整政府工作定位，全面树立“有所为，有所不为”、“该为的一定为好，不该为的一事不为”的施政理念。继续深化政府机构改革，将原有的11个行业办精简为1个，保留39人，分流107人。巩固行政审批制度改革成果，行政审批、核准、审核、备案事项减少到301项。

　　致力建设服务政府。进一步加强了政务服务中心的建设；设立了经济服务热线“89110”，共受理查处破坏经济发展环境案件913起；举办了全市银企营销洽谈会，授信金额达107亿元，为全市经济发展注入了活力；集中清理了137个党政机关单位的办公用房，共清理盘活闲置办公用房19栋，面积达41845平方米；先后两次公开拍卖处理党政机关和财政全额拨款单位的24台超标车辆；政府17个工作部门认真接受了行风评议或进行自评，服务水平进一步提高。

　　致力建设责任政府。制定实施《株洲市人民政府工作规则》；颁布施行了《株洲市行政机关工作人员行政过错责任追究暂行办法》和《株洲市行政审批监督管理办法》，明确审批责任，规范审批行为；大力推行价格（收费）公示制，切实减轻企业和群众负担。充分发挥行政监察和审计监督职能，严肃工作纪律，严明工作责任，有力地促进了廉政建设；组织清理国家公职人员拖欠银行贷款2600多万元，清偿率为100%，增强了政府公信力。

　　致力建设法治政府。认真执行人大及其常委会的决议、决定，自觉接受人大及其常委会以及人民政协和社会各方面的监督。全年共办理人大代表建议175件，政协委员提案233件，答复率、见面率均为100%，满意率达到99.8%。制定出台《关于加强和推进依法治市工作意见》，进一步增强了公务员的法制观念；认真对待和妥善处理群众来信来访；继续深入开展“严打”整治斗争，治安形势相对平稳；充分发挥工商、税务、物价、统计、路政、文化、药监、海关、招投标、检验检疫、质量技术监督等行政职能作用，打击各种不法行为，整顿和规范了市场经济秩序；切实加强安全生产工作，减少了重大事故发生。

　　各位代表，2003年在全球经济波动加剧、地区竞争日趋激烈的大背景下，面对“非典”疫情和旱涝灾害的冲击，我市经济社会保持了快速协调发展。这是市委正确领导，市人大、市政协支持监督，各民主党派、工商联、各人民团体参政议政和各界朋友热情帮助的结果，是全市人民齐心协力，沉着应对各种复杂局面，开拓进取的结果。在此，我代表市人民政府，向全市广大干部群众，向各级人大代表和政协委员，向人民解放军驻株部队、武警官兵，向所有关心和支持株洲发展的社会各界人士，表示衷心的感谢和崇高的敬意！

　　同时，我们也清醒地看到，我市经济社会发展中仍然存在不少困难和问题，主要表现在：电力、煤炭等资源约束矛盾比较突出；新兴产业发展迟缓，部分国有企业生产经营困难；就业和再就业压力较大，农民和部分城镇居民增收缓慢；环境污染问题没有得到有效解决；一些部门和单位服务意识不强、办事效率不高，等等。对于这些问题，我们将进一步采取有效措施，切实加以解决。

　　二、明确奋斗目标，实现经济社会发展新跨越

　　本届政府任期跨越国家“十五”和“十一五”两个“五年计划”时期。在研究衔接产业发展规划工作中，我们尤其感到，实现株洲发展新跨越，必须在继续坚持全市经济发展战略和基本思路的基础上，突出产业的支撑作用和核心地位。为此，我们进行了广泛的调查研究和深入的分析论证，初步制定了到2010年全市产业发展的远景规划：用7年左右的时间，通过大力推进产业开放和改革调整，整合资源，发挥优势，壮大实力，力争到2010年全市GDP突破900亿元，三次产业比调整为8:54:38，把株洲建成我国中部地区先进制造业基地和区域性商贸物流中心；建成交通装备制造、有色金属冶炼及深加工、化工、陶瓷、农产品加工、现代物流业等6大产值过百亿的支柱产业，重点培育信息、旅游、医药等3个新兴产业；发展壮大株冶、电力机车厂、硬质合金集团、时代、南方、湘火炬、株化、唐人神、千金药业、国光等10大企业集团；建设城市轨道车辆制造基地、太子奶乳酸菌制品等10大重点工程；按照政策引导、产业集群、配套发展的原则，以城区为圆心，形成“三圈”产业布局，即以发达的现代服务业为主体的中心城区服务业经济圈；以栗雨、清水塘、田心、宋家桥、董家塅为主体的沿快速环道工业组团经济圈；以白马、龙头铺、白关、渌口、马家河等城镇为结点的城市周边卫星城镇经济圈。

　　紧扣市十二届人大一次会议确定的本届政府任期目标，即到2007年，将我市基本建成全国文明城市、国家园林城市、国家卫生城市、国家环保模范城市、中国优秀旅游城市，破解事关经济与社会发展全局的10大问题，围绕经济发展和社会进步的10大事项，我们将全力办好10件大事：1、积极做好前期工作，全力争取进入国家改造振兴老工业基地的“笼子”。2、通过产业发展规划和产业政策的引导，培育壮大一批产值过亿元、5亿元、10亿元、50亿元的企业集团。3、加强水利设施建设，确保旱涝保收面积在80%以上；启动洮水水库建设。4、启动建设醴潭、衡炎、田黄三条高速公路，改造提升醴陵城至株洲县伞铺、攸县新市至株洲县朱亭、炎陵县沔渡至金竹山公路三条骨干支线，完善“三纵十二横”的骨干公路网络，打造“株洲三小时、县市一小时”的快速交通圈；积极争取武广铁路客运专线在株洲布线设站，衡茶吉铁路立项。5、全面建成总投资20亿元、总里程32公里的城市快速环道，再建两座湘江大桥，形成城市内外两环；建设城区段湘江生态经济带。6、加大环境保护力度，加强“两港一湖”（白石港、建宁港和天鹅湖）和“三废”治理，重点抓好清水塘地区环境综合整治和农村生态体系建设。7、按照“省市共建，以省为主”的方针，组建在校本科生3万人左右，拥有河东、河西两个校区的湖南工业大学。8、完善市疫病控制体系，通过招商引资，在河西建设一座标准较高的综合性医院。9、建成总投资4亿元的市体育中心，迎接2006年省十运会在我市举行。10、建设好文化园三期工程、株洲大剧院、炎帝陵二期等一批城乡文化标志性工程。

　　今年政府工作的指导思想是：认真贯彻党的十六届三中全会和市委九届十次全会精神，牢固树立“五个统筹”的科学发展观，坚定不移地实施经济和社会发展的“四大战略”，继续突出深化国有企业改革、发展非公有制经济、扩大对外开放三个经济工作重点，加快推进“三化”进程，促进经济社会全面协调快速发展。全市经济社会发展的主要预期目标是：实现国内生产总值423亿元，比上年增长11%；财政总收入和一般预算收入均增长11%；城镇居民人均可支配收入增长9%，农民人均纯收入增长5%；城镇登记失业率控制在5%以内，人口自然增长率控制在5.7‰以内；城市化水平提高到41.5%；各项事业都有新的进步。

　　在新的一年，我们将在促进经济社会快速发展的同时，注重改善城乡居民生活质量，努力做好与群众生活密切相关的10件实事：

　　1、搞好城镇“低保”和农村救济工作。对城镇“低保”户做到“应保尽保”，覆盖面达到100%；建立农村特困户救济制度，按每人每月不低于5元的标准发放，覆盖面提高到100%。

　　2、实施城乡安居工程。全年发放住房公积金个人贷款1亿元以上，启动拆迁安置房和农民转城安置房建设，完成1000户农村特困户的安居房建设。

　　3、推进就业和再就业工程。积极创造条件，力争新增城镇就业岗位4.2万个，培训失业人员2万人，再就业人数达到2.2万人。同时，新增劳务输出人员5万人。

　　4、加强社区建设。完善社区服务功能，改善市民生活居住环境，为“企业人”向“社会人”转变创造条件。同时，帮助社区居委会改善工作条件。

　　5、加快城乡电力、自来水网改造步伐。逐步实现企业生产、生活水电分离、城镇居民“一户一表”，基本实现城乡用电同网同价。

　　6、完善城市配套设施。在城市人流密集区增加建设15个公共厕所；新增若干个垃圾转运站；完善一批城市公园，所有公园实行免费开放；建成市医疗废弃物处理中心。

　　7、继续实施食品药品放心工程。加快农村药品供应网络和监督网络建设，力争“两网”覆盖70%以上乡镇；让城乡居民吃上“放心肉”、“放心菜”。

　　8、加强医疗卫生体系建设。在全市137个乡镇建立预防保健站，抓好农村中心卫生院建设，搞好农村新型合作医疗制度试点；城镇社区卫生服务覆盖面力争有更大提高。

　　9、巩固“普九”成果。设立100万元专项基金，扶助城乡贫困学生；多渠道筹集资金1500万元，加快农村学校信息化、全市教育网的建设。

　　10、实施平安株洲工程。整合各类资源，建立110、120、12315、12358等联动的市民求助服务系统，为市民提供全天候紧急报警、医疗救助、投诉受理等便捷服务；启动消防特勤站建设，进一步完善消防设施。

　　三、突出产业发展，掀起经济建设新高潮

　　加快经济发展，是全面建设小康社会的物质基础，是解决前进中一切困难和矛盾的根本出路。面对全国各地竞相发展的新形势，我们必须以改革的大突破激发经济活力的大迸发，以产业的大发展推动经济的大跨越，努力把经济增长潜力最大限度地挖掘出来，促进国民经济持续快速健康发展。

　　致力推动经济发展。突出抓好城区与县域产业发展规划和产业布局，努力构建农业高产高效、工业先进发达、第三产业繁荣有序的产业发展新格局。

　　做好“三农”工作，促进县域经济发展。高度重视并认真解决农业、农村和农民问题，重点在这几个方面努力：以实施种苗工程为基础，提高农业科技应用水平。积极推广新品种和新技术，推进农产品安全检测标准体系和质量认证体系建设，提高农产品市场竞争力。以壮大农产品加工业为突破口，推进农业产业化进程。加强规模生产基地建设，大力发展特色经济作物和草食动物，支持一批农产品加工企业做大做强，延伸产业链条，提高农业产业化程度。以项目开发为着力点，加快农村工业化步伐。依托矿产、小水电、劳动力等资源优势，精心包装一批项目，引进各类资本和技术，提高县域经济外向度和工业化水平。以加强服务引导为支撑，大力发展劳务经济。坚持“积极引导、搞好服务”的工作方针，不断拓宽劳务输出渠道，引导和鼓励更多的农民工回乡创业。

　　坚持“三个”结合，促进工业经济振兴。坚持结构调整与资本营运相结合，发展壮大企业集团。按照“有进有退，退是为了更好的进”的思路，在深化市属国有企业改革的同时，鼓励支持骨干企业发挥品牌、资金、技术、管理等方面的优势，打破行业、地域、所有制界限，通过资本营运，实现低成本扩张。坚持产业发展与项目建设相结合，实施项目带动战略。按照“经济发展长远抓规划，近期抓项目”的思路，加大项目开发力度，争取国家和省里在国债、国家创新基金、省高新技术产业引导资金方面的支持；全力支持系列航空发动机研制及生产线技改等一批新上项目启动实施；支持轨道车辆制造、高纯金属产业化等一批在建项目达产达效。坚持产业集群与园区建设相结合,形成特色产业区域。按照“招商兴园、以园兴工”的思路，充分发挥园区的聚集、辐射和带动效应，以高新区和醴陵陶瓷工业园为重点，大力发展园区经济，引导企业向专业园区和特色城镇集中，形成产业集群。

　　强化“三个”意识，促进第三产业繁荣。强化精品意识，提升市场档次。按照新的产业规划布局，继续组织好芦淞市场群的“两改一提”，积极推进汽车超市等重点工程。强化创新意识，搞活商贸流通。引导推行连锁经营、物流配送、电子商务、代理制等现代流通方式，配套发展租赁市场、耐用消费品市场，构筑社会化的现代流通体系。支持扩大住房、汽车等消费信贷，进一步激活房地产市场，培育新的消费热点。强化开放意识，发展旅游产业。进一步完善旅游发展规划，重点开发建设炎帝陵、桃源洞、云阳、酒埠江、大京、空洲岛、仙庾岭等旅游景点，开发工业旅游和观光休闲农业，做大做强旅游产业,争创中国优秀旅游城市。按照改革创新的思路，大力发展金融、证券、保险、邮政、通讯、信息、律师、会计等服务业。

　　继续深化各项改革。一是农村各项改革。以《农村土地承包法》为指导，按照“依法、自愿、有偿”原则，规范农村土地使用权流转；按照“减轻、规范、稳定”的总要求，建立规范高效的税收征管体制，取消农业特产税，逐步降低农业税；按照“放开、搞活”的方针，全面推进农村水利设施产权和经营体制改革。二是国有企业改革。继续推进以股份制改造和“置换国有资产，有偿解除职工劳动关系”为主要形式的产权制度改革，积极支持中央、省属大中型企业实行“主辅分离，辅业改制”。三是城建投融资体制改革。加大经营城市力度，多渠道筹集建设资金；按照“投建分离，市区共建”的思路，加快城市主次干道的改造步伐。四是行政管理体制改革。继续深化行政审批制度改革，不断改进政务服务中心工作。进一步理顺市县两级财政管理体制，加大依法治税和财源建设的力度；稳步推进会计集中核算向国库集中支付过渡，逐步健全公共财政体制。坚持“建管分离，监管分开，块块为主，条条监督”的方针，大力推进城市管理体制改革，相对集中行政处罚权。按照脱钩、分类、放开、搞活的原则，大力精简机构和人员，全面创新事业单位管理体制。

　　全面扩大对外开放。以加快招商引资为突破口，形成生产要素聚集的“洼地”。抓住国际资本和沿海发达地区产业加速转移的机遇，以企业、园区为主体，以项目开发为基础，以代理招商、网络招商、敲门招商、以商招商为手段，鼓励引进外资、民资、技术和人才。以扩大外贸出口为重点，大力发展外向型经济。认真研究和应对国家调整出口退税政策的影响，进一步实施以质取胜、科技兴贸和出口多元化战略，创新贸易方式，搞好协调服务，增强“株洲产品”的市场竞争力。以参与国际经济分工和合作为着眼点，加快实施“走出去”战略。鼓励和支持优势企业到境外投资办厂和承包工程，在更大范围和更深程度上参与国际经济技术合作。

　　四、基础设施先行，拓展经济社会发展新空间

　　加强城乡基础设施建设，是保持国民经济持续快速发展的基本前提，是提升区域经济综合实力的重要保障。我们将坚持在科学规划的基础上，大力进行旧城改造和新城拓展，加大交通、能源和城乡基础设施建设力度，实现城乡发展空间的大拓展，人居环境的大优化。

　　继续加强城市基础设施建设。围绕“东提西拓，合拢三角”的城市发展思路，按照第六次城市总体规划修编和全市土地利用总体规划修编的要求，制定河东三区拓展规划，拉开城市骨架，扩大城市容量。突出抓好“一带、一港、一湖、两环、四街、六路”建设。“一带”即启动湘江生态经济带建设。“一港”即完成建宁港芦淞区段治理改造，“一湖”即治理天鹅湖。“两环”包括城市外环和内环，外环是指城市快速环道，实现东湖立交竣工，加快建宁大桥建设进度，启动西环线、南环线建设；内环是指由建设路、长江路、芦淞大桥、白石港大桥构成的中心区内环道，年内启动芦淞大桥建设，做好白石港大桥的前期准备工作。“四街”即支持城区共建好四条样板街。“六路”即加快株洲大道建设进度，建设庐山路、建宁大道、新塘路、金山路，改造建设北路（二期工程）。完善城市绿化规划，依法拆除15万平方米违法建设；新建一批街头绿地，建成荷塘、天池等一批城市公园。加快铁西路等旧城改造项目的实施进度。

　　继续加强农村基础设施建设。抓住国家加强县城电网改造、国家开发银行贷款项目向县市延伸的机遇，突出产业支撑，强调功能配套，以县城扩容提质为重点，建成规模适度、布局合理、经济繁荣、特色鲜明的城镇群体。加快农田水利基本建设步伐，全面完成水利建设“5553”工程，实现“十百千万”目标，即完成各类水利工程项目5万处，搬移土石方5千万方，投入劳动工日5千万个，投入水利建设资金3亿元；整修10公里湘江防洪大堤，初步建成城市防洪四个闭合圈，治理100座病险水库，配套防渗清淤1000公里渠道，整治10000口水塘。加强农业生态环境建设，做好植树造林、退耕还林、荒山治理和水土保持工作，全市退耕还林14.5万亩，森林覆盖率达到56.9%。抓好农业综合开发，努力把开发项目建成农业现代化示范工程。

　　继续加强交通能源建设。加快醴潭高速株洲段建设，完成莲易高等级公路株洲段、106国道醴陵北段和茶陵至炎陵段的改造任务，搞好沔渡至金竹山公路提质改造；做好衡炎、田黄两条高速，醴陵城至伞铺、新市至朱亭两条骨干支线的建设前期工作；全面完成328公里县到乡（镇）公路建设，正式启动惠及166个村的“通达工程”。支持建好株洲航电枢纽工程，加快“天然气入株”项目实施进度；建成河西220KV变电站，新建两座110KV变电站；以小水电开发、沼气利用为重点，加快农村生态能源建设。

　　五、发展各项事业，构建人与自然和谐发展新格局

　　在社会主义市场经济条件下，政府的一项重要职责，就是坚持以人为本，发挥公共财政职能，大力发展各类社会事业，落实三项基本国策，以事业的全面进步，实现人的素质全面提升，以国策的全面落实，推动可持续发展的顺利实现。

　　注重体系建设，努力提高科技创新能力。抓住我市被列入“国家区域技术创新工程试点城市”的机遇，以企业研发能力建设为核心，重点加强5个国家级、7个省级技术中心和工程研究中心的建设，提升5个企业博士后工作站的科研实力，构筑科技创新体系。力争将创业服务中心和中小企业促进园建成省内一流的孵化器，构建科技中介服务平台和体系，抓好科技成果转化和知识产权保护。建立风险投资和信贷担保体系，增加高新技术产业投入。

　　强化载体支撑，大力发展各项社会事业。巩固“普九”成果，扩大高中教育规模，稳步发展中等职业教育，积极调整教育资源，加快湖南工业大学建设步伐。健全突发公共卫生事件应急机制，完善疾病控制等公共卫生服务体系。加快体育中心场馆建设进度，积极开展群众体育活动。启动市影剧院等文化设施的改造，加大文化产业开发力度，继续搞好“周周乐”等广场文化活动，丰富人民群众生活。与此同时，我们将把人才队伍建设放在重中之重的位置来抓，创新人才工作机制，完善人才市场体系，唯才是举、唯才是用，大力培育和引进高层次、高技能人才，构筑株洲人才新高地。

　　关心群众生活，进一步完善社会保障体系。千方百计抓好就业和再就业工作，落实再就业扶持政策，加强就业技能培训，拓宽就业渠道，扩大就业规模。进一步完善和健全社会保障体系，做好养老保险扩面和改制企业续保工作，完善失业保险和医疗保险体系，继续实施工伤保险和生育保险，落实城市居民最低生活保障制度。贯彻落实住房公积金管理条例，扩大公积金缴存面。高度关注弱势群体，发展各类慈善事业，做好扶贫和救灾工作，健全社会福利、社会救济、优抚安置救助体系，支持老年人、残疾人事业，加强法律援助工作，关心城乡特困户生活。

　　落实基本国策，继续实施可持续发展战略。抓好计划生育工作，大力发展妇女儿童事业，控制人口增长，提高人口素质。依法保护耕地，实现耕地总量动态平衡，稳步推进国土与矿产资源市场化进程。以创建全国环保模范城市为目标，加大“三废”和噪声治理力度，重点抓好清水塘地区环境综合整治，力争市区空气质量进一步好转，饮用水源水质达标率96%以上。

　　六、切实转变职能，促进政府自身建设跃上新台阶

　　进一步加强公共管理，加快转变政府职能，提高干部队伍素质，努力把政府建设成为精干、务实、高效的政府，亲民、爱民、恤民的政府，不缺位、不越位、不错位的政府。

　　——加强公共管理，提高政府工作效率。充分发挥政府各有关部门的职能作用，加强市场监管，大力打击各类欺行霸市、制假售假、涉税违法、贩黄贩非、规避招投标等行为，整顿和规范市场经济秩序。继续整顿社会信用秩序，努力创建金融安全区，营造诚信为本的金融环境。坚持不懈地开展“严打”整治斗争，加强社会治安综合治理，创造平稳可控的治安环境。搞好城市维护管理，努力改善市容市貌，营造整洁舒适的人居环境。充分发挥“经济89110”的作用，严肃查处影响和破坏我市经济发展环境的行为。加强安全生产监督，抓好消防与道路交通安全检查，防止各种重特大安全事故发生。推进统计管理体制改革，加快区域统计运行步伐。

　　——加强作风建设，提高政务服务水平。大力开展行政效能监察，建立以办事时限制为重点的七项工作制度，进一步明确工作标准和工作要求，对行政机关不作为、缓作为、乱作为等行政过错，加大监察和责任追究力度。贯彻实施《行政许可法》，加强政府法制工作，提高依法行政水平。充分利用互联网、热线电话等方式建立社情民意反映制度。建立与群众利益密切相关的重大事项社会公示和听证制度，不断完善专家咨询制度和民主化决策程序。

　　——加强队伍建设，提高公务员整体素质。坚持严格标准、公平竞争、择优录用、德才兼备的原则，做到凡进必考，严把“录用关”。大力开展教育培训，努力建立一支知识化、年轻化、专业化的公务员队伍，严把“素质关”。规范办事程序，严格奖惩措施，做到有章可循、有法可依，严把“管理关”。继续深入开展廉政建设和反腐败斗争，旗帜鲜明地鼓励有为者，批评空谈者，惩治腐败者，严把“廉政关”。

　　各位代表，准确把握好政府的角色定位，是做好政府工作的基本前提。我们将自觉坚持党的统一领导，全面实施市委的决策部署；认真执行人民代表大会及其常委会的决议和决定，尊重人民政协、各民主党派、工商联以及其他社会各界人士的意见，加强与法院、检察院、军队、武警等国家机关以及工会、共青团、妇联等群众团体的联系；真诚接受民主监督、法律监督和人民监督、社会监督有效结合的全方位监督；认真做好人民群众来信来访工作，帮助他们解决工作和生活中的实际困难。

　　各位代表！全面建设小康社会，任务光荣而艰巨。让我们在市委的坚强领导下，解放思想，与时俱进，励精图治，开拓创新，为加快株洲的经济社会发展，创造株洲更加美好的未来而努力奋斗！
